# Acute Zika Virus Infection after Travel to Malaysian Borneo, September 2014

**DOI:** 10.3201/eid2105.141960

**Published:** 2015-05

**Authors:** Dennis Tappe, Stephan Nachtigall, Annette Kapaun, Paul Schnitzler, Stephan Günther, Jonas Schmidt-Chanasit

**Affiliations:** Bernhard Nocht Institute for Tropical Medicine/World Health Organization Collaborating Centre for Arbovirus and Haemorrhagic Fever Reference and Research, Hamburg, Germany (D. Tappe, S. Günther, J. Schmidt-Chanasit);; University Medical Center Heidelberg, Heidelberg, Germany (S. Nachtigall, A. Kapaun);; University of Heidelberg, Heidelberg (P. Schnitzler);; German Centre for Infection Research, Hamburg (S. Günther, J. Schmidt-Chanasit)

**Keywords:** Zika virus, hearing disorder, Borneo, travel, viruses, Germany, Zika fever

**To the Editor:** Zika virus (ZIKV), a mosquito-borne flavivirus, causes Zika fever, a self-limiting febrile and exanthematic arthralgia syndrome closely resembling dengue fever. Most often, signs and symptoms are maculopapular rash, fever, arthralgia, myalgia, headache, and conjunctivitis; edema, sore throat, cough, and vomiting occur less frequently (*1*). The virus, which was initially isolated from a rhesus monkey (*Macaca mulatta*) in 1947 in Uganda, has come to attention recently after a large outbreak occurred in the western Pacific region, including French Polynesia, New Caledonia, Easter Island, and the Cook Islands (*2*). Travel-related imported infections have thus been increasingly reported from the western Pacific and sporadically also in travelers to other regions of the world, including Thailand, Indonesia, and Senegal (*2**,**3*). ZIKV is transmitted by different *Aedes* mosquito species, and nonhuman primates play a role as reservoirs (*1*). After the beginning of the ZIKV epidemic in late 2013, a 20-fold increase of Guillain-Barré syndrome incidence was noted in French Polynesia; 1 patient was infected a week before neurologic symptoms started (*4*). We report an acute ZIKV infection in a traveler returning from Malaysian Borneo who experienced bilateral hearing difficulties during the course of illness.

On September 1, 2014, a 45-year-old woman was seen in an outpatient clinic in Heidelberg, Germany for fever of up to 39°C and maculopapular rash covering her trunk, arms, and legs. Fever had started on August 30, which was 6 days after she had returned from a 3-week vacation to peninsular Malaysia and Sabah, Malaysian Borneo. Laboratory analyses showed a slightly elevated C-reactive protein level of 5.2 mg/L (reference range <5.0), but liver function test and complete blood count results were within reference range. During the next 3 days, the fever subsided, but the patient experienced a sore throat, bilateral conjunctivitis, and a burning sensation of the palms and soles. These symptoms were accompanied by swelling of the hands and increasing arthralgia of the wrists, palms, and fingers. There was no lymphadenopathy. An indirect immunofluorescence assay for ZIKV (*3*) demonstrated an IgM titer of 1:640 and an IgG titer of 1:320 (cutoff <1:20) on day 6 of illness (Figure). An indirect immunofluorescence assay for dengue virus demonstrated an IgG titer of 1:80 and no IgM (cutoff <1:20). 

**Figure:**
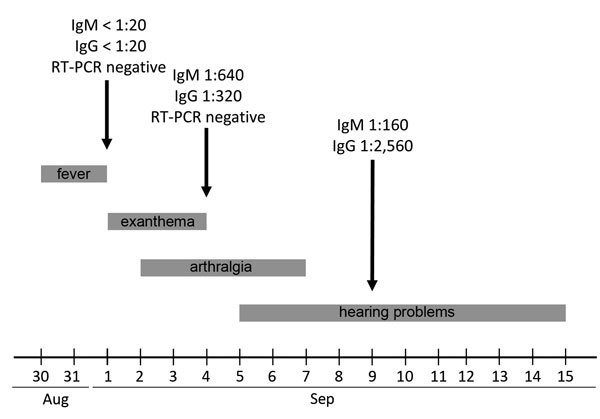
Clinical course and laboratory results (reverse transcription-PCR [RT-PCR]) for a patient with Zika virus (ZIKV) infection acquired from Malaysian Borneo.

Two days later, the patient experienced sudden bilateral dull and metallic hearing; in her left ear, she experienced a very short delay between a sound and her perception of the sound. Follow-up ZIKV serologic testing on day 11 of illness showed a decreased IgM titer of 1:160 and an increased IgG titer of 1:2,560 (Figure). Viral neutralization testing (*3*) of the same sample demonstrated the presence of ZIKV-specific neutralizing antibodies. Chikungunya virus serology results were negative. An archived serum sample from day 3 of illness studied by ZIKV serology and a ZIKV-specific real-time reverse transcription PCR (*3*) was negative (Figure). Hearing difficulties lasted for 10 days and resolved gradually (Figure). 

During her journey to several cities and villages in Sabah, Malaysian Borneo, the patient had noticed several mosquito bites even though she had used repellents. She had stayed in hotels, private homes, and remote church homes under various conditions (Technical Appendix).

In Asia, Zika fever has been described sporadically in Cambodia, Thailand, and Indonesia (Java and Lombok) (*1**,**3**,**5**,**6*). On the basis of the incubation time of ≈6 days in returning travelers (*2**,**3*), we assumed that the patient became infected in Keningau or surrounding villages, in northern Borneo. Although ZIKV was detected in *Ae. aegypti* mosquitoes in peninsular Malaysia in 1969 (*7*) and antibodies against ZIKV were demonstrated in serum samples from 15 of 79 patients on peninsular Malaysia and 9 of 50 patients in Borneo in 1953 (*8*), Zika fever in peninsular Malaysia or Borneo has not been reported. In 2001, ZIKV seropositivity was demonstrated in a native Bornean, 2 migrants to Borneo, and 2 Bornean orangutans (*Pongo pygmaeus*) (*9*). A later study found an additional 8 Bornean orangutans to be seropositive for antibodies against ZIKV (*10*). Thus, in Borneo, either the virus only rarely infects humans or the disease is mistaken for dengue fever. 

Neurologic complications of ZIKV infections had previously been reported only as Guillain-Barré syndrome, and hearing difficulties in Zika fever patients have not been reported. Because this symptom resolved spontaneously, no audiometry or auditory brainstem response testing was performed, and the cause of the disorder remains unclear. Because of increasing travel and migration and heightened clinical and laboratory awareness, more ZIKV infections are likely to be diagnosed outside of epidemic events.

Technical Appendix Travel itinerary of a patient with Zika fever.
