# Supplementary material for: Acute Zika Virus Infection after Travel to Malaysian Borneo, September 2014
Source: Emerg Infect Dis. 2015 May;21(5):911–3. doi: 10.3201/eid2105.141960 (PMC4412240; doi:10.3201/eid2105.141960)
Supplement: Technical Appendix — Travel itinerary of a patient with Zika fever. [file 14-1960-Techapp-s1.pdf]

DOI: http://dx.doi.org/10.3201/eid2105.141960

Acute Zika Virus Infection after Travel to Malaysian Borneo, September 2014

Technical Appendix

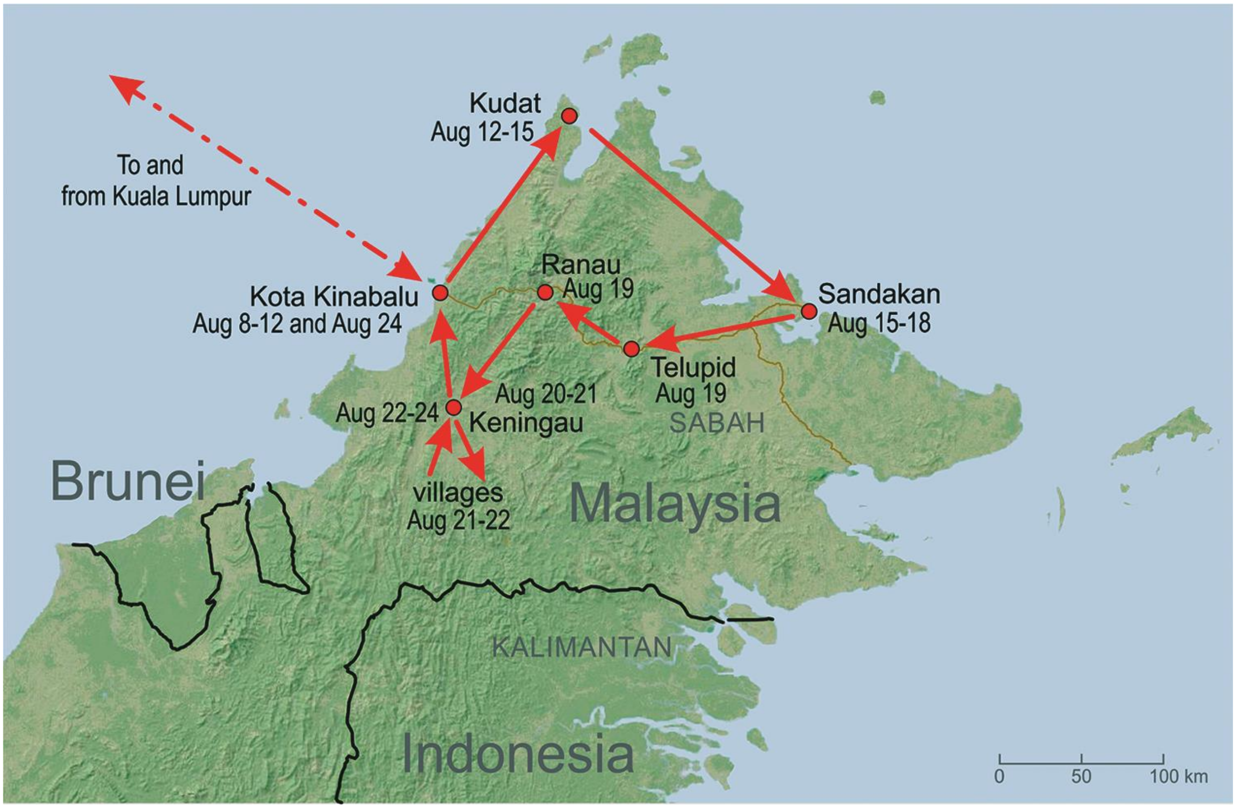

Technical Appendix Figure. Map of northern Borneo showing the itinerary through the Malaysian state of Sabah of a patient with Zika fever. The patient most likely became infected while staying in Keningau and southern villages (Papalungan), where she also took a boat trip on the Sungai Papalungan, a river through the rain forest.
